# Genomic hallmarks of parasexual reproduction in three hybrid groups of the human pathogen *Cryptococcus neoformans*

**DOI:** 10.64898/2026.05.28.728413

**Published:** 2026-05-31

**Authors:** Rahul Anand, Qinxi Ma, Diana Tamayo, Grace Paul, Nicolas Helmstetter, Sheng Sun, Zhuyun Bian, Kyung J. Kwon-Chung, Joseph Heitman, Rhys A. Farrer

**Affiliations:** 1 Medical Research Council Centre for Medical Mycology at the University of Exeter, Department of Biosciences, Faculty of Health and Life Sciences, Exeter EX4 4QD, United Kingdom; 2 Department of Molecular Genetics and Microbiology, Duke University Medical Center, Durham, North Carolina, 27710, USA; 3 Molecular Microbiology Section, Laboratory of Clinical Immunology and Microbiology, National Institute of Allergy and Infectious Diseases, National Institutes of Health, Bethesda, Maryland, USA

## Abstract

Hybridization is a major driver of fungal evolution, yet knowledge of the molecular mechanisms underpinning hybridization and its genomic impact remain limited. Here, we analyse 197 *Cryptococcus neoformans* genomic sequences, including 13 newly sequenced strains, identifying three genetically clustered and distinct hybrid groups (H1, H2 and H3) each with unique parental origins and ecological associations. Using phylogenomics, population structure analyses, and long-read genome assemblies, we identified hybrid genomes with chromosome-wide loss of heterozygosity (LOH), inheritance of large intact parental haplotype blocks and widespread aneuploidy within and across genomes. These patterns are also observed when progeny were generated with *spo11*Δ parents, indicating these features are a result of a meiotic-independent process such as parasex. This is further underscored by discovery of haploid and near-haploid recombinants in both *spo11*Δ mutant progeny and reanalysed the wild-type hybrid progeny. We hypothesise these haploid and near-haploid recombinants are generated through ploidy reduction via independent chromosome assortment because of concerted chromosomal loss, which is a key feature of the parasexual cycle. Phenotypic assays demonstrated that several hybrid isolates have diverse growth and virulence patterns, underscoring functional consequences of genome plasticity. Together, our work suggests a non-meiotic reproductive process contributes to shape the genotypic and phenotypic diversity of *Cryptococcus neoformans*.

## Introduction

Fungal species propagate through a diverse array of strategies, encompassing both sexual and asexual modes (1). Sexual reproduction occurs via meiotic and/or mitotic recombination process during outbreeding (heterothallism) or selfing (homothallism) (1). In heterothallic fungi, mating is initiated by the recognition of compatible partners through mating type-specific peptide pheromones and receptors, followed by cell-cell fusion. Genetic exchange typically requires compatibility at the mating-type loci (*MAT***a** and *MAT*α), which can be organised as a single genomic loci (bipolar) or across multiple loci (e.g., tripolar, tetrapolar) (2). This process culminates in the formation of spores, which facilitate dispersal and long term survival through dormancy, stress resistance, and the ability to colonise new environments (3, 4).

While meiosis is the hallmark of canonical sexual reproduction, some fungi also engage in parasexual reproduction this non-meiotic process that includes genome fusion, mitotic recombination, and ploidy reduction resulting in chromosomes originating from either parent (5–7). First described by Guido Pontecorvo in *Aspergillus nidulans* (8), parasexuality has since been reported in multiple fungal phyla including other Ascomycota species such as *Candida albicans* (6, 7), *Fusarium oxysporum* (9) and *Verticillium dahlia* (10). Evidence of parasexual cycles have been found in the Basidiomycota genus *Ustilago* (11), and in the Chytridiomycota amphibian pathogen *Batrachochytrium dendrobatidis* (12).

*C. neoformans*, *C. deneoformans*, and the *C. gattii* species complex are important human pathogens, together responsible for over 180,000 deaths annually worldwide (13). The sexual cycle of the human-pathogenic fungus *Cryptococcus neoformans* was first described in 1975 by June Kwon-Chung (14). Sexual reproduction occurs between *MAT***a** and *MAT*α cells, which fuse to form dikaryotic hyphae. Mating is influenced by environmental cues such as inositol or nutrient limitation, which has been documented in V8, bird guano, and decaying wood (4, 15, 16). At the tips of the hyphae, a basidium forms in which nuclear fusion and meiotic recombination occur, giving rise to infectious basidiospores (17). Despite having both mating types, over 98% of clinical and environmental isolates of *C. neoformans* are *MAT*α (18, 19), suggesting that *C. neoformans* primarily propagates via asexually or via unisexual (same-sex *MAT*α) reproduction (18, 20). Under appropriate nutrient conditions (MS or V8 media), *MAT*α cells can produce basidiospores independently, whereas *MAT***a** cells typically do not - potentially contributing to the global predominance of the *MAT*α genotype (21). However, a recently published paper demonstrate congenic *MAT****a*** and *MATα* were both equally well able to undergo unisexual reproduction (22).

In Europe and parts of the Americas, *Cryptococcus* hybrid isolates - particularly those between serotype A *C. neoformans* (VNI) (previously known as var. *grubii*) and serotype D *C. deneoformans* (VNIV) (previously known as var. *neoformans*) account for up to 30% of clinical cases (23), while estimates from high-burden regions such as Africa and Asia suggest hybrids account for closer to 10% of cases (24). The discovery that these AD hybrids are diploid, typically having opposite mating types revealed they are produced through mating of haploid cells of the two sister species *C. neoformans* and *C. deneoformans* (25–27). While many AD hybrid strains are no longer self-fertile, a few have been identified that retain the ability to produce monokaryotic hyphae, basidia and spores. Spore dissection revealed a low (~5%) spore germination rate, and the spores that germinated produced progeny found to be AD diploid with no evidence of meiotic reduction to the haploid state. Thus, the genetic differences between the two parental lineages appear to preclude meiotic recombination and chromosomal reassortment. These hybrids can exhibit altered phenotypes, including enhanced virulence (28), reduced antifungal susceptibility (29), and increased resistance to ultraviolet light (30), impacting epidemiology and clinical outcomes. Natural hybrids have been recovered from both clinical and environmental sources. Laboratory-generated crosses have revealed extensive genomic plasticity following hybridization including aneuploidy, chromosomal rearrangements, and LOH (24, 31, 32). However, most studies have focused on VNI × VNIV crosses, overlooking the potential for hybridization involving other *C. neoformans* lineages such as VNII, VNBI, or VNBII (33). Therefore, the broader genomic and phenotypic consequences of hybridization remain poorly understood.

To investigate the genetic diversity and evolutionary history of *C. neoformans*, we performed phenotypic profiling and whole genome sequencing for 13 isolates that were analysed alongside 184 publicly available genome sequences. We identified three distinct *Cryptococcus neoformans* hybrid groups and resolved their parental lineage origins. We uncovered diverse genomic signatures of hybridization - including significantly larger genome sizes, aneuploidy, whole chromosome LOH and F_ST_ changes, and haplotype inheritance patterns that are hallmarks of parasexual reproduction, a mode of recombination not previously documented in *Cryptococcus*. Through generating the first ever scaffold-level phased genome assemblies of 4 hybrid isolates, we reveal the genome structure of hybrid genomes, corroborating our parasex hypothesis. Next, by generating and crossing *spo11*Δ mutants of both *C. neoformans* H99 (VNI) and *C. deneoformans* JEC20 (VNIV), we recovered similar genomic patterns, suggesting a non-meiotic Spo11-independent pathway for recombination, consistent with parasex. Hybrid isolates exhibited phenotypic variation in traits such as virulence and growth rate, highlighting functional consequences of hybridization. We suggest that parasex is a key driver of genome evolution, population structure, and phenotypic diversity in *C. neoformans*.

## Results

**Figure 1:**
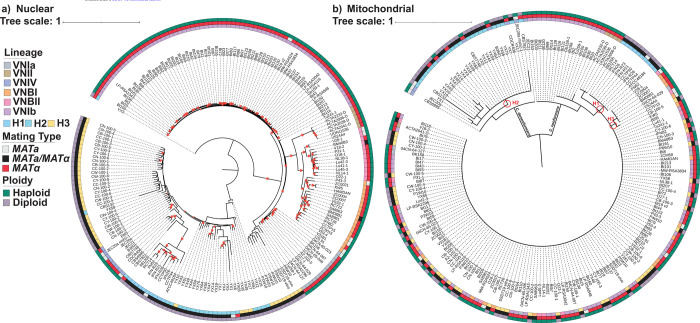
Phylogenetic analysis suggests 3 cryptococcal hybrid groups between VNBI and VNIV (H1), VNI and VNIV (H2 and H3). Phylogenetic trees for 197 *C. neoformans* isolates were constructed for the nuclear genome (**a**) and mitochondrial genome (**b**). Phylogenetically informative sites were calculated by Phylorust, where positions are covered ≥ 90% of isolates.

A nuclear phylogeny inferred from 61,284 phylogenetically informative sites across 197 *C. neoformans* isolates resolved six major monophyletic clades corresponding to *C. neoformans* lineages (VNIa, VNIb, VNII, VNBI, VNBII) and *C. deneoformans* (VNIV) (Fig. 1a; Table S1). Hybrid isolates were distributed across nuclear phylogeny rather than forming a single clade, which is consistent with mixed ancestry and multiple hybrid origins. Notably, a subset of clinical hybrid isolates formed a distinct nuclear cluster, suggesting shared ancestry of recent divergence. In contrast, the mitochondrial phylogeny revealed three well-supported hybrid groups (H1-H3, with nodes highlighted with red) (Fig. 1b). H1 hybrids clustered with VNII/VNBI (*C. neoformans*), H2 hybrids clustered within the VNIV lineage (var. *neoformans*), and H3 hybrids clustered with VNI (*C. neoformans*), indicating lineage-specific mitochondrial inheritance. Comparing the nuclear and mitochondrial phylogenies revealed extensive discordance (Fig. S1), consistent with hybridisation followed by backcrossing. Together, our phylogenetic analyses support at least three independent hybridisation events

**Figure 2:**
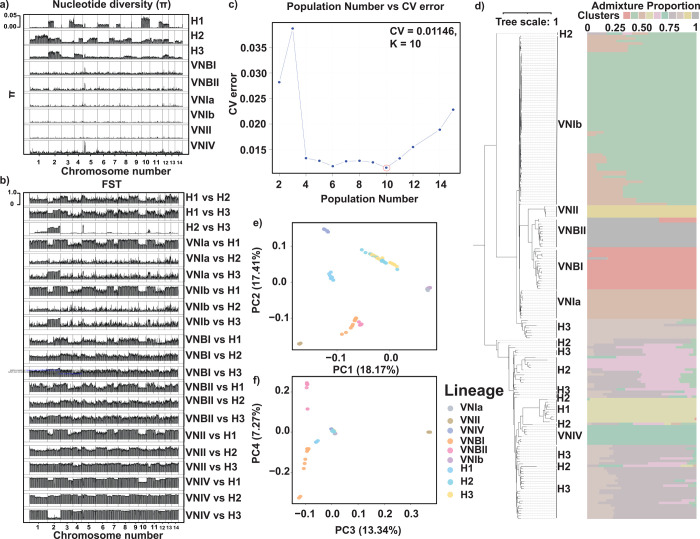
Population genomics reveals differences in nucleotide diversity, FST, and admixture between hybrid groups. **(a)** Nucleotide diversity (π) plotted across the genome for six distinct *C. neoformans* lineages and three hybrid groups. **(b)** Pairwise genetic differentiation (F_ST_) plotted across the genome, comparing the hybrid groups to each other and to the six lineages. **(c)** Cross-validation (CV) error from unsupervised ADMIXTURE analyses across K values; the lowest CV error (0.01146) occurs at K = 10. **(d)** ADMIXTURE ancestry proportions for all isolates at K = 10, showing mixed ancestry in VNIb, H2 and H3, whereas VNIa, VNII, VNBI, VNBII and H1 show little to no admixture. **(e, f)** Principal component analysis (PCA) of genomic variant sites, with isolates clustering by lineage. H1 forms a distinct cluster compared with H2 and H3. The first four principal components (PC1–PC4) together explain 56.19% of the total variance, with PC1 and PC2 shown in **(e)** and PC3 and PC4 shown in **(f)**.

Genome-wide nucleotide diversity (π) across non-hybrid isolates was uniformly low (VNIa π = 0.002; VNIb π = 0.001; VNBI π = 0.005; VNBII π = 0.004; VNIV π = 0.005), whereas all hybrid groups display substantially elevated diversity (H1 π = 0.007; H2 π = 0.012; H3 π = 0.008) (Fig. 2a). Plotting π across 10 kb sliding windows reveals pronounced heterogeneity across hybrid genomes, with regions of elevated diversity with interspersed tracts of low diversity. We found that mitochondrial π is uniformly low across all hybrid isolates (Fig. S2), further highlighting discordance between nuclear heterogeneity and mitochondrial uniformity.

Genome-wide F_ST_ profiles reveal minimal divergence between H2 and H3 (mean F_ST_ = 0.07), with both groups showing high divergence from H1 (H1 vs H2 mean F_ST_ = 0.41; H1 vs H3 mean F_ST_ = 0.51) (Fig. 2b). In contrast, mitochondrial F_ST_ values are uniformly high when comparing hybrid groups (Fig. S2b) (H1 vs H2 mean F_ST_ = 0.69; H1 vs H3 mean F_ST_ = 0.96; H2 vs H3 mean F_ST_ = 0.88), reinforcing lineage-specific mitochondrial inheritance.

Unsupervised ADMIXTURE analysis of nuclear SNPs shows K = 10 being the best fitting model (K = 10; CV = 0.01146) (Fig. 2c), resolving distinct non-hybrid lineages and revealing varying degrees of admixture among hybrid groups (Figs. 2d and S3). H2 and H3 show a highly admixed ancestry profiles, whereas H1 formed a distinct cluster with minimal admixture. Principal component analysis (PCA) corroborated these patterns, with H1 forming a discrete clustered whilst H2 and H3 overlapping with parental lineages (Fig. 2e-f). TriangulaR was used to characterise hybrid classes by plotting hybrid index against interclass heterozygosity (Fig. S4). Several isolates, particularly within H2 and H3, clustered near hybrid index of ≈ 0.5 and heterozygosity ~ 1.0, consistent with progeny being recent F1 hybrids. However, most hybrids deviated from this expectation, exhibiting reduced heterozygosity and shifts in hybrid index towards one parental lineage. This is consistent with LOH, resulting in reduced heterozygosity, whilst shifts in hybrid index consistent with uniparental chromosome inheritance or chromosome loss. Notably H1 hybrids showed greater deviation in hybrid index from F1 expectations, which may be a result of backcrossing to a parental lineage, extensive LOH and chromosome loss, consistent with reduced admixture and a distinct population structure.

Mitochondrial population structure within the isolates analysed was markedly simpler (K = 4; CV = 0.00395) (Fig. S2c). We identified a clear divide between isolates belonging to *C. deneoformans* (VNIV), which forms a single unadmixed population, and *C. neoformans* (VNI, VNBI, VNBII and VNII), which forms two populations with evidence of admixture between the two populations (Fig. S2d). Surprisingly, our ADMIXTURE analysis suggested that H1 hybrids form a distinct unadmixed mitochondrial population, while H2 hybrids fall within *C. deneoformans* and H3 hybrids fall within *C. neoformans* populations. Similarly, PCA analysis using mitochondrial SNPs also identified H1 hybrids forming a separate group compared to the other isolates analysed (Figs S2e-f).

*MAT*α is the most common mating type (57.6%), while *MAT***a**/*MAT*α was identified in 36% of isolates, and *MAT***a** found in only 6.60% of the isolates (Table 1). We found that most hybrid isolates have both mating types present (*MAT***a**/*MAT*α: 93.24%; *MAT***a**: 2.70%), suggesting that unisexual hybridisation is rare (28).

**Figure 3:**
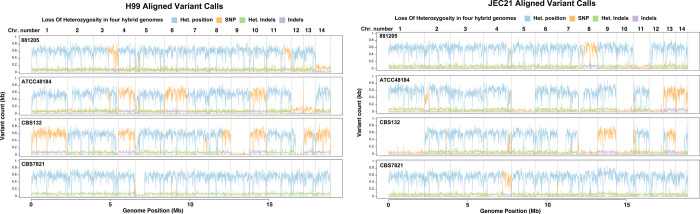
LOH and long runs of homozygosity seen throughout hybrid genome. Variant counts plotted across the genome with (blue: heterozygous positions; orange: homozygous SNPs; green: heterozygous indels; purple: homozygous indels) across 4 hybrid isolates (**a**) 881205, (**b**) ATCC48184, (**c**) CBS132 and (**d**) CBS7821 aligned to both H99 (*C. neoformans*) and JEC21 (*C. deneoformans*) reference genomes.

We identified hybrid genomes exhibit an abundance of heterozygosity across each hybrid genome, interrupted by large tracts of homozygous regions (Figs. 3 and S5). Tracts of loss of heterozygosity (LOH) ranged from localised segments to whole chromosome. Some tracts of LOH also appear to be localised to putative chromosome arms (e.g., on chr3 and chr11 in isolate 881205). LOH was identified in each of the three hybrid groups. However, the distribution of LOH varied substantially among isolates. Large contiguous LOH tracts are inconsistent with canonical meiosis alone instead being indicative of mitotic recombination such as observed as part of the parasexual cycle observed in other fungi. In addition, the variability of LOH patterns across isolates is consistent with ongoing genome restructuring, suggesting hybrid genomes are dynamic rather than static.

To further resolve the genomic architecture of hybrid isolates, phased haplotype assemblies of 4 hybrid isolates were generated using long read oxford nanopore (ONT), and each separated into 2 haplotypes and a single mitochondrial sequence (Table S2; Fig. S6). The resulting assemblies revealed marked variation in haplotype length variation across isolates, with haplotypes ranging from 17.83 Mb (haplotype 2 CBS132) to 22.067 Mb (haplotype 2 ATCC48184).

**Figure 4.**
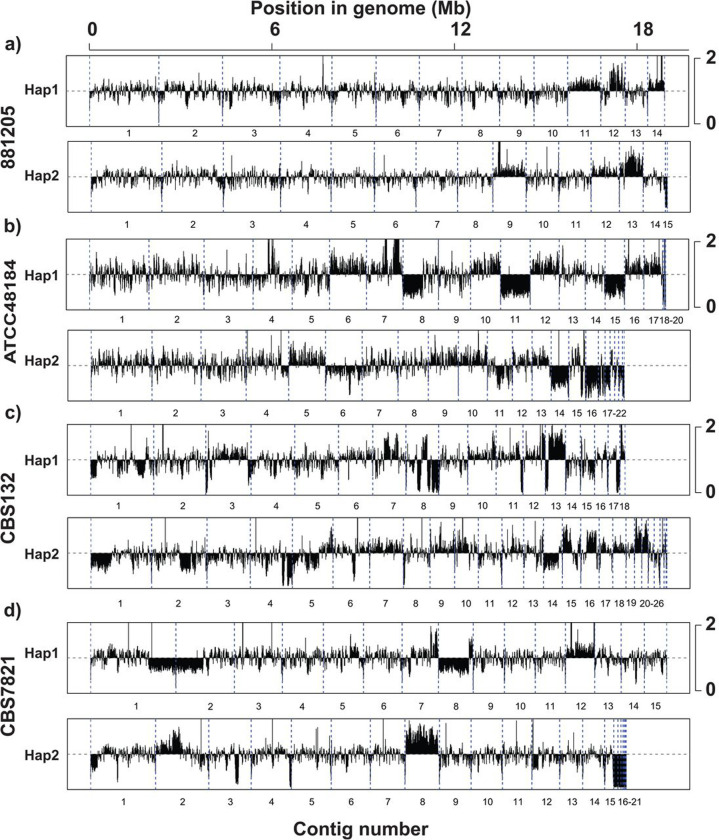
Aneuploidy and evidence of copy number variation was identified in each hybrid genome. Normalised depth of coverage plotted across haplotypes of the phased cryptococcal hybrid genome assemblies (**a**) 881205, (**b**) ATCC48184, (**c**) CBS132 and (**d**) CBS7821. Segmental aneuploidy can be seen through the genomes of all hybrid cryptococcal isolates in a “stair step” structure with regions of increased coverage flanked with normal or reduced coverage.

Haplotype-specific ploidy and copy number variation is revealed through plotting depth of coverage and tally of percent of reads agreeing the reference base across haplotypes (Fig. 4; Fig. S7). This divergence is further supported by low shared sequence identity between haplotypes (Fig. S8; Table S3). Synteny analysis comparing two haplotypes to each other and their parental lineages (*C. neoformans* and *C. deneoformans*), reveals haplotype 1 is highly syntenic to *C. neoformans* whilst haplotype 2 is highly syntenic to *C. deneoformans* (Fig. S9). Together, these observations indicate substantial structural divergence between homologous chromosomes, potentially limiting recombination between parental haplotypes.

**Figure 5:**
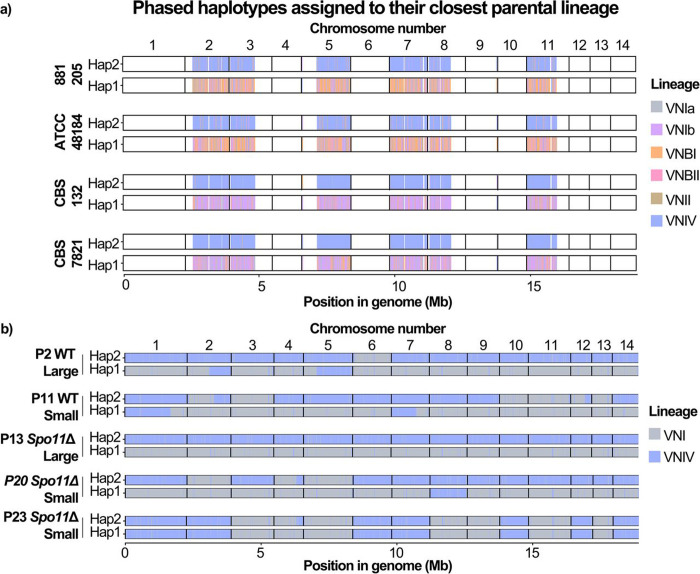
Parental-Lineage Haplotype Windows Across Hybrid Genomes. **A)** Haplotype pairs of positions that are phased in 4 hybrid isolates (881205, ATCC48184, CBS132 and CBS7821) plotted across 10kb windows, and haplotypes coloured to their closest relative (grey: VNIa, purple: VNIb, orange: VNBI, brown: VNII, blue: VNIV, white: genomic regions that are not phased in all, and therefore not directly comparable). **B)** Haplotypes pairs for lab crossed hybrids, across ‘phased in any’ sites, plotted across 10kb windows, with haplotypes coloured to their closest relative across 5 isolates (AD hybrid Progeny (P)2 Wild type (WT) Large, P11 WT Small, P13 Spo11Δ Large, P20 Spo11Δ Small and P23 Spo11Δ Small haploid progeny.

Phased haplotypes were assigned to their closest parental lineage by phylogenetics, across 10kb windows (Figs. 5a and S10, Table 2). Haplotype 2 in all hybrids show to be predominantly derived from the VNIV parent, whereas haplotype 1 showed variable contributions from VNIb or VNBI depending on the isolate. Parental ancestry was structured in large segmental and unbroken blocks, rather than smaller interspersed blocks, indicating limited recombination breakpoints, inconsistent with canonical meiosis and rather indicates mitotic recombination and chromosomal reassortment.

To quantify recombination, pairwise comparison of overlapping phase positions was calculated. We first used a positive control (pairwise comparison of phased *Aspergillus fumigatus*; highest percentage of crossovers identified = 0.41) (Table S4) and negative control (pairwise comparison of *Candida albicans*; highest percentage of crossovers identified = 0.02) (Table S5). The number of crossovers varied substantially between pairwise comparisons, with the highest being 393 (between 881205 and CBS7821) and the lowest being 11 (between 881205 and ATCC48184) (Table S6). In both cases, crossovers corresponded to a small fraction of phased positions. We classified a small number of those crossovers as potentially meiotic (crossovers that are > 1 kb apart from any other crossover and have > 2 phased positions either side of the crossover supporting the phasing). The highest number of potentially meiotic crossovers detected were between 881205 and CBS7821 (n = 21) and the lowest detected was between 881205 and ATCC48184 (n = 3). Overall, we identified potential meiotic crossover events occurring at a low frequency through hybrid genomes. This data, combined with parental haplotypes being inherited in large segmental blocks is inconsistent with hybridisation occurring via canonical-meiosis, rather supporting a mitotic recombination process which leads to extensive haplotype preservation.

To assess phenotypic variation, we tested the growth of eight hybrid isolates and four reference isolates (two each from *C. neoformans* (VNI) and *C. deneoformans* (VNIV) in three media types: YNBGlu, RPMI, and YPD (Fig. S11a–c). The *C. deneoformans* reference strain JEC21 exhibited the slowest growth in all conditions. In contrast, *C. neoformans* (H99) and *C. deneoformans* (B3501) isolates showed the fastest growth overall. Among hybrids, ATCC48184 had the highest growth in YPD and RPMI, while 881205 performed best in YNBGlu. CBS132 exhibited the slowest hybrid growth in both YPD and RPMI and was second slowest in YNBGlu. Therefore, we found no evidence for a characteristic growth rate of hybrids compared to non-hybrids. Virulence for the hybrid group isolates was evaluated using a *Galleria mellonella* infection model (Fig. S11d). *C. neoformans* H99 was the most virulent strain, causing 0% larval survival by day 6. In contrast, non-hybrid strains 1CM50 (*C. neoformans*), JEC21 (*C. deneoformans*), and B3501 (*C. deneoformans*) were significantly less virulent, with some larvae surviving beyond 10 days. Hybrid isolates showed wide variation in virulence: CBS132 and CBS7821 were highly virulent, resembling H99 with complete larval mortality by days 6 and 7, respectively. In contrast, 881205 and ATCC48184 exhibited attenuated virulence, with notable larval survival through the 10-day assay. Therefore, hybrid isolates do not show evidence of increased virulence, but they may have other untested advantages to non-hybrids.

To test whether the pattern of haplotype inheritance and mosaic structure of hybrid genomes is meiosis-independent, we analysed progeny derived from bilateral *spo11*Δ crosses. Spo11 is required to initiate double-stranded breaks (DSB) during meiosis, so *spo11*Δ mutants would have impaired meiosis during hybridisation. These crosses provide a means for us to test whether the genome patterns seen in hybrids are dependent on canonical Spo11-mediated meiosis. Interestingly, we observed a modest increase in spore germination frequency in the *spo11*Δ bilateral crosses (27%) compare to wild-type crosses (9.5%), which suggests that Spo11 is generating breaks during reproduction between *C. neoformans* and *C. deneoformans*, but these breaks are not leading to productive meiotic recombination, presumably because the DNA mismatch repair system is aborting the crossover events due to the genetic divergence in the genomes of the two parental strains. When Spo11 is mutated, these non-productive DNA breaks are no longer generated, leading to higher overall spore germination rates, although the underlying cause of this increase in germination rates cannot be precisely resolved from the present data alone.

Both wild-type and *spo11*Δ progeny showed evidence of aneuploidy and copy number variation across several chromosomes, consistent with other hybrid genomes analysed within this paper (Fig. S12). Aneuploidy was most pronounced across small progeny, showing triploid, diploid and haploid chromosomes. Whereas large progeny showed more uniform diploidy and depth of coverage across their genomes. Strikingly, *spo11*Δ progeny 23 allele frequency distributions across the genome are broadly consistent with a largely haploid genome. Even more strikingly, half of the chromosomes appeared to be monomorphic with the *C. neoformans* H99 parental strain, whereas the other half of the chromosomes were monomorphic with the *C. deneoformans* JEC20 parent (Fig. 5b). This pattern of inheritance suggests independent chromosomal assortment in the absence of meiotic recombination. However, we do note that, normalised depth of coverage plots revealed some modest localised increases and decreases in coverage, indicating underlying copy number variation despite the overall haploid signal. Therefore, genomic restructuring through aneuploidy and copy number variation during hybridisation occurs even when meiotic recombination is impaired. Moreover, the phenotype-dependent patterns of chromosome instability are consistent with genome remodelling through meiosis-independent processes, such as parasexual concerted chromosomal loss.

Plotting haplotype ancestry revealed similar proportions of parental inheritance across each of the progeny. Three isolates (P2 WT large, P13 *spo11*Δ large and P23 *spo11*Δ small) have near equal proportions of VNI and VNIV (~50:50) (Fig. 5b). However, 2 progenies had a slight skew towards VNI (P11 WT small = 59% and P20 *spo11*Δ small = 58%). In both WT and *spo11*Δ progeny, haplotypes were inferred in large segmental blocks, as phased SNP data revealed extended genomic regions with consistent parental assignment and limited allele switching. For each of the hybrid assemblies, haplotype 1 was mostly derived from the *C. neoformans* parent while haplotype 2 was mostly derived from the *C. deneoformans* parent (Fig. S13). Pairwise comparison between overlapping phase positions from hybrid (and diploid) progeny revealed low numbers of crossovers (Fig. S14). The highest number of crossovers identified by any comparison was 13 through a comparison between P3 and P6 WT large. This low level of crossovers observed in both WT and *spo11*Δ progeny suggests hybridisation is not dependent on meiosis and instead may be caused by mitotic recombination which can occur during parasex.

To determine if this reduction in ploidy results in haploid recombinant AD hybrids, we reanalysed sequencing data of 20 progeny reported by Kwon-Chung and Varma (2006) (34), including isolates previously classified by FACS as haploid recombinants. Plotting normalised depth of coverage (Fig. S15a) demonstrates copy number variations (CNVs) throughout the hybrid genomes. Plotting tally of percentage of reads agreeing with base (Fig. S15b) revealed 8 isolates as either predominantly or completely haploid. When plotting variants across the hybrid genomes we demonstrate LOH and LROH patterns seen in the other hybrid isolates analysed in this study (Fig. S16). We see LOH sometimes coinciding with a reduction in ploidy, such as that seen in chromosome 3 of isolate SB-8. These observations are consistent with concerted chromosome-loss, and independent chromosome assortment, a feature of parasexual reproduction in which hybrid progeny lose ploidy to generate a recombinant reduced progeny cell.

## Discussion

High-throughput sequencing has refined our understanding of *Cryptococcus neoformans* phylogenomics and population structure, enabling identification of globally prevalent clades and lineage-specific variation linked to virulence (35, 36). Our study uncovers unexpected complexity within *C. neoformans* hybrids, previously grouped as a single category (23). Phylogenetic analysis reveal at least three genetically distinct groups, diverging through their differences in parental contributions, as seen through our haplotype analysis, and patterns of mitochondrial inheritance confirming previous hypothesise of *C. neoformans* hybrids resulting from multiple independent hybridisation events (26).

Through characterisation of hybrid genomes, including those originating from experimental crosses, we find evidence that hybridisation is meiosis-independent and instead, that genomes have signatures of a parasexual (or similar) cycle. Phylogenetic and haplotype analyses reveal three distinct hybrid groups (H1, H2 and H3) defined by differing parental contributions and mitochondrial phylogenetic groupings, confirming multiple independent hybridization events. We see divergence in population structure between hybrid populations, H1 hybrids exhibit minimal admixture - consistent with either a single sexual event, or backcrossing to a single parental lineage, while H2 and H3 hybrids shows extensive admixture and a stratified population structure. Potential H1 backcrossing may promote genomic homogenization and enhance fitness, as observed in other fungal hybrids such as *Microbotryum* (37).

*Cryptococcus* hybrids are highly aneuploid, consistent with prior reports of genomic plasticity in *C. neoformans* hybrids (38). We identified distinct copy number variation (CNV) patterns, including a “stair-step” structure where a central region of elevated CNV flanked by normal or reduced coverage, mirroring rearrangements linked to antifungal resistance in *Candida albicans* (39). Frequent whole-chromosome CNVs, as supported by ploidy variation between haplotypes and chromosomes, suggest a parasexual cycle involving concerted chromosome loss, a mechanism known to stabilize aneuploidy and confer drug resistance in other non-meiotic fungi such as *C. albicans* (6). We hypothesize that similar parasexual processes may drive genome stabilization and adaptation in *C. neoformans* hybrids.

We identified extensive genomic mosaicism characterized by long runs of homozygosity and loss of heterozygosity (LOH) (23, 33), again consistent with parasex. Our study provides evidence of widespread LOH in naturally occurring hybrids from both clinical and environmental settings. Given that *in vivo* passaged *C. albicans* strains exhibit LOH rates 100 times higher than *in vitro* isolates (40), we suggest that LOH in *C. neoformans* hybrids may contribute to within-host adaptation and increased virulence. Varying tracts of homozygosity and heterozygosity have been observed as a key trait of parasexual reproduction in *Candida* yeast pathogens in generating diverse progeny bypassing the spore-formation process (41) and potential mechanism of rapidly acquiring antifungal drug resistance (42). We found evidence of crossovers, although haplotypes remained mostly physically segregated, paralleling patterns seen in other parasexual fungi (12). Therefore, hybrid genome structures are inconsistent with a canonical meiotic recombination pathway.

Experimental analysis of hybrid progeny derived from *spo11* deficient mutants further supports this model of a non-meiotic parasexual process of hybridisation between *C. neoformans* and *C. deneoformans*. We observed LOH, aneuploidy, evidence of concerted chromosomal loss and a pattern of large segmental haplotype block inheritance in progeny derived from *spo11*Δ mutants (which have disrupted formation of meiotic DNA double stranded breaks (43)) suggesting these processes are meiosis-independent. We also observed similar genomic structure and low numbers of crossovers in both WT and *spo11*Δ progeny, coinciding with 2 growth patterns we called large and small, reflecting their colony size.

Our findings suggest hybridisation in *Cryptococcus* might be exclusively a parasexual process, with diploid hybrid progeny predominantly remaining diploid and undergoing LOH. In some rarer cases hybrids undergo concerted chromosomal loss, as seen in progeny 23, which has a haploid genome with segmental heterozygosity only at the end of chromosome 4, corresponding to the chromosomal region involved in a translocation between the H99 and JEC21 genomes (44). Progeny 23 was identified here as an F1 progeny of *spo11*Δ mutant crosses between *C. neoformans* and *C. deneoformans* (44) as a largely haploid progeny in which independent chromosomal assortment has occurred without demonstrable meiotic recombination. In previous studies, *C. neoformans* crosses with *C. deneoformans* conducted by Kwon-Chung and Varma (2006) (34) were observed to produce some progeny that were small aneuploid or even near haploid cells. Our analysis of these F1 progeny similarly revealed independent chromosomal assortment without evidence of meiotic recombination. We note that these findings are in several ways analogous to what is observed during the parasexual cycle of *C. albicans* in which two diploid cells mate to produce a tetraploid that then undergoes concerted chromosome loss to return to the diploid state (41, 45) with only limited evidence of parameitoic recombination (46).

A key difference between the evidence of parasex in *Cryptococcus* hybrids compared with *C. albicans* hybrids, is that we have not demonstrated that that progeny that appeared haploid regained the ability to mate again, facilitating F2 hybrids along with backcrossing. However, we have some evidence of backcrossing occurring in H1 hybrid progeny, based on hybrid index analysis. Additionally, no naturally occurring *C. neoformans* or *C. deneoformans* isolates have been identified that harbour a full chromosome from the other species, and the only evidence for introgression that has been reported is an example of ancient introgression (47). Further crosses from hybrid progeny would support our hypothesis for a parasexual cycle, while hybrid sterility would suggest something distinct and resembling a ‘broken’ sexual cycle.

Our *in vitro* metabolic and *in vivo* virulence assays revealed striking phenotypic variability among hybrids. Environmental isolates CBS132 and CBS7821 showed the highest virulence in *Galleria mellonella*, suggesting environmental pressures can drive genomic changes that enhance pathogenic potential (48, 49). We propose that key adaptive mechanisms including LOH and aneuploidy arise through parasexual reproduction. These genomic traits mirror patterns in *Aspergillus nidulans*, where diploid isolates gain fitness under stress via mitotic recombination (50). Our findings suggest that similar stress-induced parasexual mechanisms in *C. neoformans* hybrids generate genomic diversity, enhancing virulence and antifungal resistance. Together, our results revealed a previously unappreciated phylogenetic and population diversity driven by a non-meiotic and potentially parasexual process, thereby impacting cryptococcal fitness and virulence.

## Materials and Methods

### DNA Sequencing

All isolates (except CBS7748 and B3501) were grown in YPD +2% glucose agar for 48h to obtain single colonies. One single colony was inoculated in YPD +2% glucose for 16h at 30°C, with shaking at 200 rpm. DNA was isolated using a combination of phenol chloroform-isoamyl-alcohol method and the Qiagen DNA mini kit (QIAmp DNA mini kit, 51304). Briefly, cells were resuspended in 200 μl of Tris EDTA buffer and 200 μl of phenol, followed by vortexing (20 sec vortex: 20 sec ice, repeated for 3 cycles). The samples were snap-frozen in liquid nitrogen, then heated to 75°C for 10 min. Next, 200 μl of chloroform:isoamylalcohol (24:1) was added and the lysate was centrifuged to separate the aqueous phase. A second chloroform:isoamylalcohol (24:1) wash was performed to remove residual phenol. The aqueous phase was precipitated with 2 volumes of absolute ethanol and incubated at −80°C for at least 2 hours. The DNA was resuspended in 400 μl of 10 mM Tris HCl pH 8.0 and treated with RNaseA (100 μg/ml) at 37°C for 30 min. DNA purification was completed using the Qiagen kit by adding 1.5 volumes of buffer AW1, followed by transfer to the DNeasy mini spin column. The column was centrifuged and washed with buffer AW2, followed by elution with 10 mM Tris HCl pH 8.0.

For isolates CBS7748 and B3501, genomic DNA was extracted from cells grown in YPD (1% yeast extract, 2% peptone, 2% glucose) and washed twice in PBS using a DNeasy Plant Pro Kit (QIAgen, #69204). Cells were lysed with 0.5 mm zirconia/silica beads in a FastPrep 24 homogenizer (MP Biomedicals) for four cycles (30s at 6m/s followed by 90s chilling on ice). All other steps followed manufacturer’s instructions.

Sequencing was performed by the Exeter Sequencing Service. Samples were prepared using the NEBNext Ultra II FS DNA Library Prep kit (NEB, #E7805) following manufacturer’s instructions. Libraries were loaded as part of a lane using XP loading on the NovaSeq 6000 using a paired-end strategy with a read length of 150 bases. All reads for these isolates have been deposited to NCBI Sequence Read Archive (SRA) under Bioproject number PRJNA1279655.

### High-molecular weight genomic DNA extraction

High-molecular weight DNA for Nanopore sequencing was extracted following Mayjonade *et al.* (2016) (51) with minor modifications. Cells were grown overnight in YPD, harvested by centrifugation, washed once with PBS, and freeze-dried overnight. The dried cell pellet was disrupted by bead-beating at 4 m/s for 10 s with 0.5 mm zirconia-silica beads using a FastPrep 24 homogenizer. A freshly prepared, pre-heated SDS-based lysis buffer (100 mM Tris-HCl pH 8.0, 50 mM EDTA pH 8.0, 0.5 M NaCl, 1% PVP40, 1% sodium metabisulfite, 5 mM DTT, 2% SDS, 200 μg/mL proteinase K and 100 μg/mL RNase A [both from 20 mg/mL stock]) was added. The sample was quickly homogenised by brief vortexing and incubated in a thermomixer at 55 °C with shaking (500 rpm, 1 h). Contaminants including proteins and polysaccharides were precipitated by adding 1/3 (vol/vol) of 5 M potassium acetate, incubating for 10 min at 4 °C, and centrifuging (8,000 × g, 10 min, 4 °C). Genomic DNA was purified from the cleared lysate using a NucleoMag^®^ DNA Bacteria kit (Macherey-Nagel, #744310.4), according to the manufacturer’s instructions starting at step 4 (DNA binding). DNA integrity was confirmed on a 0.5% agarose gel, and purity and concentration were assessed by NanoDrop spectrophotometry and Qubit fluorometry. Genomic DNA was sequenced by the Exeter Sequencing Service on the ONT PromethION using R10.4.1 flow cells with Kit 14 (E8.2) chemistry. Reads were basecalled and demultiplexed with Dorado using the super-accurate model dna_r10.4.1_e8.2_400bps_sup@v4.3.0. All reads for these isolates have been deposited to NCBI Sequence Read Archive (SRA) under Bioproject number PRJNA1322120.

### Genome assembly

ONT reads were filtered with Filtlong v0.3.1, favouring longer, higher-quality reads and downsampling to ~100× where possible (filtlong --min_length 1000 --min_mean_q 90 -target_bases 2000000000 --length_weight 10). To enable haplotype-resolved hybrid genome assembly, parental *k*-mer profiles were generated directly from the reference assemblies of *C. neoformans* H99 and *C. deneoformans* JEC21 using **yak** (v0.1) (https://github.com/lh3/yak) with “yak count -k 31 -b 32 -o out.yak in.fasta”. Hybrid assemblies were then generated with **hifiasm** (v0.25.0) (52) using Oxford Nanopore long reads and the parental *k*-mer databases to guide competitive read binning and assembly of phase-separated haplotigs: “hifiasm --ont reads.fastq.gz −1 H99.yak −2 JEC21.yak -c 1 -d 1 - o output”. For isolates CBS132 and ATCC48184, the --trio-dual option was additionally applied to improve parental haplotype balance.

Each haplotype assembly was polished using Illumina paired-end reads and **Hapo-G** (v1.3.8) (53). To assign short reads to their respective haplotypes, Illumina reads were first mapped competitively against both haploid assemblies using **BWA-MEM2** (54) (v2.2.1). The combined reference was indexed, and alignments were sorted and indexed with **samtools** (v1.22). Polishing was performed independently for each haplotype using Hapo-G e.g., “hapog --genome hap1.fasta --pe1 hap1.R1.fq.gz --pe2 hap1.R2.fq.gz -o hap1_hapog -u”. This procedure corrected residual base-level errors while preserving haplotype phasing, resulting in high-quality, polished assemblies for both parental subgenomes. The completeness of the assemblies was assessed with **BUSCO** v6.0.0 (55) using the basidiomycota odb12 lineage dataset to search for near-universal single-copy orthologs. Phasing accuracy was assessed with **yak trioeval** using parental k-mer databases (H99, JEC21), reporting genome-wide SwitchErr% (phase switches between informative sites) and HammingErr% (sites needing relabelling for consistent phase) from phase-informative k-mers across hap1/hap2.

These Whole Genome Shotgun projects have been deposited at NCBI GenBank under the accessions JBYKLF000000000 and JBYKLG000000000 (isolate 881205), JBYKLH000000000 and JBYKLI000000000 (isolate CBS7821), JBYKLJ000000000 and JBYKLK000000000 (isolate CBS132), and BYKLL000000000 and JBYKLM000000000 (isolate ATCC 48184)

### Read alignment and variant calling

In addition to the 13 isolates from our collection, we also analysed an additional 184 isolates as obtained from the NCBI SRA, selected based on the following criteria: whole-genome Illumina paired-end sequencing and taxonomic classification as *C. neoformans*, *C. deneoformans*, or *C. neoformans × C. deneoformans* hybrids. Isolates were chosen to represent all major lineages (VNI, VNII, VNB, and VNIV). We also included the MATa isolate JEC20a, as no other representative of MATa was present in our dataset. Public isolates were sourced from 10 BioProjects: PRJEB14735, PRJEB27222, PRJNA1076182, PRJNA174540, PRJNA191162, PRJNA382844 (56), PRJNA626676, PRJNA700784, PRJNA764746 (36) and PRJNA999197 (34). PRJNA700784 was included for its focus on AD hybrid isolates. Reads obtained from the NCBI SRA databases were converted to the FASTQ format using SRA Toolkit v3.0.8.

Illumina reads were aligned to 4 reference sequences: 1) *C. neoformans* H99 CNA3 (GCA_000149245.3), 2) *C. neoformans* H99 mitochondrion (CP003834.1), 3) *C. neoformans* H99 MATα locus (AF542529.2) and 4) *C. neoformans* JEC20 MATa (AF542530.2) using the Burrows Wheeler Aligner (BWA) v0.7.17-r1188 mem(54). Alignments were converted to sorted BAMs using SAMtools v1.21(57).

Variant calling was performed as previously described(58). Briefly, duplicates were marked in the alignments, and the resulting file was sorted by coordinate order. The Genome Analysis Toolkit (GATK) v.4.1.2.0(59) HaplotypeCaller was used to call variants in GVCF mode with the haploid or diploidy ploidy flag, as appropriate. Variants were imported to GATK 4 GenomicsDB and hard filtered if QualByDepth (QD) < 2.0, FisherStrand (FS) > 60.0, root mean square mapping quality (MQ) < 40.0.

### Phylogenetic and population genomics analysis

The VCF files for the 197 *C. neoformans* isolates were converted to a multi-sample FASTA using Phylorust (https://github.com/rhysf/Phylorust), using default parameters (-s 1 [homozygous SNPs only], -m 4 [min depth 4], -p [percent threshold to extract sites 90]).The resulting multi-sample FASTA file with 55441 nuclear sites per isolate. The same parameters were used for VCF files that were constructed using alignments made with CP003834.1 H99 mitochondrial reference resulting in a multi-sample FASTA file with 12 mitochondrial sites per isolates. Phylogenetic trees of both nuclear and mitochondrial FASTA files were generated using RAxML v8.2.13(60) with 1000 bootstraps and generalized time-reversible (GTR) model and category (CAT) rate approximation model with GTR-Gamma distributed rates for the final evaluation of trees. A SplitsTree(61) Bio-Neighbour Joining Network analysis was made using the same nuclear sites.

The VCF files of the 197 *C. neoformans* isolates were merged using indexed and merged using VCFtools(62) and then converted into ped and map format with plink 1.9(63). Linkage disequilibrium pruning was done on the SNPs, which then was run through an unsupervised ADMIXTURE(64) analysis with random seed (settings: -haploid=”*” and -s time) for K values 1 to 15, with K = 10 having the lowest CV error. The merged VCF file was used to calculate nucleotide diversity (π) and Hudson’s F_ST_(65) via Pixy(66).

Using SAMtools v1.21(57) BAM files were converted into SAM and MPILEUP files, which were used to calculate average depth of coverage and plotting normalised depth of coverage across 10kb windows for 10 *C. neoformans* genomes. Numbers of heterozygous and homozygous SNPs were counted across 10kb windows and plotted across the genome.

VCF and BAM files were inputted into the HaplotypeTools(12) pipeline to phase VCF files and find parental inheritance of haplotypes of *C. neoformans* genomes. Briefly the script HaplotypeTools.pl was used to phase genomes, with resulting phased VCF files inputted into the utility script VCF_phased_to_PIA.pl with setting -p 2 to find most informative phased sites in that sample and FASTA sequences of haplotype blocks/pairs using the HaplotypeTools utility script VCF_phased_and_PIA_to_FASTA.pl. HaplotypePlacer.pl was used to find the nearest relative of each hybrids by iteratively constructing phylogenetic trees using FastTree(67) to find nearest relative for each haplotype.

### Antifungal resistance assay and growth curves

All 13 strains were grown overnight in YPD at 30 ˚C with shaking. Cells were collected by centrifugation at 4,000 rpm for 5 min, washed twice with PBS, and adjusted to OD_600_=0.02 in YPD, RPMI1640 (supplemented with 2% glucose and 0.165 M 3-N-morpholinopropanesulfonic acid (MOPS), pH=7), or YNBGlu (2% glucose, 0.65% yeast nitrogen base without amino acids). OD_600_ was read hourly by a Tecan M-Plex plate reader at 30 ˚C for 48 h. The mean of four independent measurements was determined for each strain. Area under the curve (AUC) was calculated by using GraphPad Prism (version 10.2.1).

Virulence assay was performed in the invertebrate model *Galleria mellonella*. *C. neoformans* strains were grown overnight in YPD at 30 ˚C with shaking. Cells were inoculated to YPD from overnight cultures were inoculated to 10 ml YPD to OD_600_ = 0.2 and grown for 4 h at 30 ˚C. Cells were collected by centrifugation at 4,000 rpm for 5 min, washed twice with PBS, and adjusted to 1×10^7^ cells/ml in PBS. Each larva was injected with 10 μl of fungal cell suspension (10^5^ fungal cells) containing 10 μg ampicillin and 3 μg kanamycin through the last pro-leg on the left. PBS containing antibiotics was included as control. 20 larvae were infected per strain. The larvae were then incubated at 37˚C in the dark and survival quantified for 10 days post-infection.

### Crosses involving *spo11*Δ deletion mutants and analyses of random spore

To analyse the effect of Spo11 during hybridization between *C. neoformans* and *C. deneoformans*, we conducted two types of crosses: H99 (*C. neoformans*, wild type) × JEC20 (*C. deneoformans*, wild type) and H99 *spo11*Δ::*NAT* × JEC20 *spo11*Δ::*NAT*, and recovered random spores by microdissection following protocols published previously (68, 69). Spore germination rates were calculated and then 6 large and 6 small colonies from each cross were patched, from which genomic DNA was purified and sequenced at the Duke Sequencing and Genomic Technologies core facility (https://genome.duke.edu), on a Novaseq X platform with 250 bp paired-end option.

## Supplementary Material

Supplement 1

Supplement 2

Supplement 3

Supplement 4

Supplement 5

Supplement 6

Supplement 7

## Figures and Tables

**Table 1:** High prevalence of hybrids heterozygous at the *MAT* locus. Mating type percentages of each cryptococcal lineage/hybrid group.

variety	Lineage	MATa	MATa/MATα	MATα
grubii	VNBI	23.53%	5.88%	70.59%
	VNBII	16.67%	8.33%	75.00%
	VNIa	8.33%	0.00%	91.67%
	VNIb	1.45%	0.00%	98.55%
	VNII	0.00%	0.00%	100.00%
	all	6.96%	1.74%	91.30%
neoformans	VNIV	37.50%	0.00%	62.50%
hybrid	H1	10.00%	90.00%	0.00%
	H2	4.35%	82.61%	13.04%
	H3	0.00%	100.00%	0.00%
	all	2.70%	93.24%	4.05%
all	all	6.60%	36.04%	57.36%

**Table 2:** Parental contributions to hybrid genomes. Haplotype clade summary of hybrid *C. neoformans* isolates, with percentage of genome inherited from each respective lineage.

C. neofomans					C. deneoformans
solates	VNIa	VNIb	VNBI	VNII	VNIV
881205	5.83	11.56	32.04	0.61	49.96
ATCC48184	3.35	14.98	31.13	0.62	49.92
CBS132	7.51	37.87	4.01	0.62	49.99
CBS7821	9.93	35.11	4.44	0.56	49.97

## Significance statement

Hybridisation in fungal pathogens has been linked to novel genotypes, some of which have enhanced virulence. *Cryptococcus* is a human pathogen responsible for approximately 180,000 deaths annually worldwide. However, the mechanisms by which *Cryptococcus* generates genotypic diversity remains understudied. By analysing 197 *C. neoformans* genome sequences, we identified three distinct groups of hybrids, defined by their ancestral inheritance. In each of these 3 groups, we discovered novel features of hybrid isolates including uneven chromosome numbers in most but not all hybrids, chromosome-wide loss of heterozygosity consistent with whole chromosome inheritance from a single parent, and whole-chromosome-wide haplotype inheritance. These genomic features are consistent with a parasexual form of reproduction, which is a non-meiotic process involving cell and nuclear fusion, followed by gradual chromosome loss and sometimes mitotic recombination. Surprisingly, similar genomic patterns were observed in *spo11*Δ mutant crosses, supporting the hypothesis that a non-canonical process such as parasex is facilitating cryptococcal hybridisation. Our results expand our understanding of fungal reproduction and highlight new routes for the emergence of virulence and antifungal resistance.
