# Supplementary material for: Genomic hallmarks of parasexual reproduction in three hybrid groups of the human pathogen Cryptococcus neoformans
Source: bioRxiv. 2026 May 31:2026.05.28.728413. Preprint. [Version 1] doi: 10.64898/2026.05.28.728413 (PMC13308457; doi:10.64898/2026.05.28.728413)
Supplement: Supplement 1 [file media-1.docx]

**Supplemental Information**

**Genomic hallmarks of parasexual reproduction in three hybrid groups of the human pathogen *Cryptococcus neoformans***

Rahul Anand^1^, Qinxi Ma^1^, Diana Tamayo^1^, Grace Paul^1^, Nicolas Helmstetter^1^, Sheng Sun^2^, Zhuyun Bian^2^, Kyung J. Kwon-Chung^3^, Joseph Heitman^2^, Rhys A. Farrer^1^.

^1^Medical Research Council Centre for Medical Mycology at the University of Exeter, Department of Biosciences, Faculty of Health and Life Sciences, Exeter EX4 4QD, United Kingdom

^2^Department of Molecular Genetics and Microbiology, Duke University Medical Center, Durham, North Carolina, 27710, USA

^3^Molecular Microbiology Section, Laboratory of Clinical Immunology and Microbiology, National Institute of Allergy and Infectious Diseases, National Institutes of Health, Bethesda, Maryland, USA

**Supplementary Table Legends**

**Table S1.** Details of variant calling of 197 isolates analysed in this study using GATK.

**Table S2.** Variable lengths of *C. neoformans* long-read assemblies. Hybrid assemblies consisting of haplotype 1, haplotype 2 and mitochondrial sequences were assembled.

**Table S3.** Alignment percentages of haplotypes when compared to each other of hybrid phased assemblies.

**Table S4.** Pairwise comparison of overlapping phased positions in *Aspergillus nidulans* phased isolates, representing a positive control of meiotic crossover identification

**Table S5.** Pairwise comparison of overlapping phased positions in *C. albicans* phased isolates, representing a negative control of meiotic crossover identification

**Table S6.** Pairwise comparison of overlapping phased positions in *C. neoformans* hybrid isolates showing crossover counts within the genome

**Supplementary Figures**

**Figure S1 Discordance revealed between nuclear and mitochondrial phylogenies.** Tanglegram comparing the nuclear SNP phylogeny (left) and mitochondrial SNP phylogeny (right) for the same 197 isolates. The extensive mismatch in isolate ordering between the two trees indicates cytonuclear (nuclear–mitochondrial) discordance, consistent with hybridisation and subsequent recombination/backcrossing. Hybrid isolates separate into three mitochondrial-defined groups (H1–H3): H1 associates with var. *grubii* lineages VNII/VNBI, H3 associates with VNI (var. *grubii*), and H2 associates with VNIV (var. *neoformans*).

**Figure S2 Mitochondrial population genetic analysis. (a)** Nucleotide diversity (π) plotted across the mitochondrial genome. **(b)** Hudson F_ST_ values plotted across the mitochondrial genome. **(c)** Unsupervised admixture analysis using mitochondrial SNP sites revealed a population size of 4 (K=11; CV error = 0.00395). **(d)** Admixture plot, show 4 distinct largely unadmixed mitochondrial populations, with var. *grubii* consisting of two populations, whilst var. *neoformans* consists of 1 population. Interestingly H1 hybrids show a distinct mitochondrial population, with no evidence of admixture. **(e,f)**, Principal component analysis (PCA) of mitochondrial SNPs similarly demonstrate H1 hybrids forming a distinct cluster, apart from the rest of the hybrids and non-hybrid lineages.

**Figure S3** ADMIXTURE plot with labels from the nuclear genomes of 197 isolates of C. neoformans, K=10.

**Figure S4 TrianglulaR plots of hybrid index versus interclass heterozygosity indicate predominantly F1 hybrids.** Plots generated using triangulaR based on ancestry-informative markers (AIMs) (63) show hybrid isolates relative to parental lineages. (**a**) H1 (VNBI x VNIV); (**b**) H2 (VNI x VNIV) and (**c**) H3 (VNI x VNIV). Isolates clustering near hybrid index ≈ 0.5 with a high level of heterozygosity ~ 1.0 are consistent with F1 hybrids, whereas a lower heterozygosity suggests isolates belonging to later generation hybrids.

**Figure S5 LOH observed across hybrid genomes.** Variants plotted across genome with (blue: heterozygous positions; orange: homozygous SNPs; green: heterozygous indels; purple: homozygous indels) across *C. neoformans* genomes, including 12 hybrids from each representative hybrid group identified in our analysis (1: VNI; 4: H1; 4: H2; 4: H3).

**Figure S6** Mitochondrial maps.

**Figure S7 Ploidy variation seen across phased long read hybrid assemblies.** Alleles frequencies (percent of reads to agreeing with the reference base) plotted from 25% agree to 75% disagree. Red-dotted lines that indicate the highest support for diploidy are at 46-53% whilst lines at 30-36% and 63-69% show greatest support for triploidy. These frequencies plotted from contigs 1-6 with 4 *Cryptococcal* hybrid genomes.

**Figure S8 Dot plots reveal divergent nature of hybrid haplotype sequences.** Dot plot aligning haplotype 1 and 2 from phased ONT long-read hybrid assemblies. Black is sequences with < 25% identity; brown < 50%; dark green < 75% identity; light green > 75% identity.

**Figure S9.** **Synteny between the 2 haplotypes of hybrid assemblies and reference genome assemblies, showing the location of the MAT locus (blue circle) and STE when not flanking the MAT locus (orange circle). A)** Synteny between reference genome assemblies. **B)** Synteny between haplotypes of phased assemblies and parental reference genomes of H99_3 (*C. neoformans*) and JEC21 (*C. deneoformans*).

**Figure S10 Phylogenetic trees of largest haplotypes reveal segregation of parental haplotypes.** Phylogenetic tree of 2 of the largest haplotypes found (**a**) chromosome 11, locus 486151-489972 which is 3821 bp long and (**b**) chromosome 5, locus 850607-853699 which is 3092 bp long.

**Figure S11** **Phenotypic and virulence profiles for hybrid isolates.** Phenotypic assays assess growth and virulence in eight *C. neoformans* isolates, including four hybrids. Black = VNI, blue = VNIV, orange = hybrids. (**a-c**) Growth curves and area under the curve (AUC) comparisons are shown for YPD, RPMI, and YNB+glucose conditions. * indicates difference compared to *C. neoformans* H99 (**d**) Virulence was evaluated in *Galleria mellonella*, with survival plotted across time.

**Figure S12 Aligning *C. neoformans* hybrids crosses to *C. neoformans* H99 reveal copy number and ploidy variations throughout both WT and spo11Δ progeny** Percent of reads agree plotted across chromosomes (**a**) demonstrate near-uniform diploidy in both WT

**Figure S13 Phylogenetic analysis of largest haplotypes reveals segregation of haplotypes in both WT and spo11Δ** **crosses.** Phylogenetic tree of 2 of the largest haplotypes (chromosome 6:1000958-1006704; chromosome 11:486082-492538) of phased hybrid progeny (progeny 2, WT large; progeny 11, WT small; progeny 13, spo11Δ large; progeny 20, spo11Δ small; progeny 23, spo11Δ small)

**Figure S14 Counts of crossovers among spo11**Δ progeny. (**a**) We classified crossovers ≥ 1kb from any other crossover and 3 ≥ phased heterozygous positions either side of crossovers as meiosis candidates. (**b**) all remaining crossovers were considered gene conversion crossovers/events.

**Figure S15 AD hybrid progeny from Kwon-Chung and Varma (2006) demonstrate ploidy variation and evidence of concerted chromosomal loss.** Alignments *C. neoformans* x *C. deneoformans* progeny to H99 *C. neoformans* reference genome used to plot (**a**) normalised depth of coverage and (**b**) tally of percent of reads agreeing to the reference base.

**Figure S16 LOH Observed across aneuploid hybrid progeny.** Variants plotted across the genome with (blue: heterozygous positions; orange: homozygous SNPs; green: heterozygous indels; purple: homozygous indels). We see large genomics regions where there is a significant drop in heterozygous positions and heterozygous indels, which sometimes are also accompanied by long runs of homozygosity (LROH).
